# Recent Progress of Nanoscale Metal‐Organic Frameworks in Synthesis and Battery Applications

**DOI:** 10.1002/advs.202001980

**Published:** 2020-12-31

**Authors:** Ming Zhong, Lingjun Kong, Kun Zhao, Ying‐Hui Zhang, Na Li, Xian‐He Bu

**Affiliations:** ^1^ State Key Laboratory of Advanced Processing and Recycling of Nonferrous Metals Lanzhou University of Technology Lanzhou 730050 P. R. China; ^2^ School of Materials Science and Engineering Tianjin Key Laboratory of Metal and Molecule‐Based Material Chemistry National Institute for Advanced Materials Nankai University Tianjin 300350 P. R. China

**Keywords:** batteries, nanoscale metal‐organic frameworks, synthesis strategies

## Abstract

As one type of promising inorganic–organic hybrid crystal material, metal‐organic frameworks (MOFs) have attracted widespread attention in many potential fields, particularly in energy storage and conversion. Recently, effective strategies have been developed to construct uniform nanoscale MOFs (NMOFs), which not only retain inherent advantages of MOFs but also develop some improved superiorities, including shorter diffusion pathway for guest transportation and more accessible active sites for surface adsorption and reaction. Additonally, their nanometer size provides more opportunity for post‐functionalization and hybridization. In this review, recent progress on the preparation of NMOFs is summarized, primarily through bottom‐up strategies including reaction parameter‐ and coordination‐assisted synthesis, and top‐down strategies such as liquid exfoliation and salt‐template confinement. Additionally, recent applications of NMOFs in batteries as electrodes, separators, and electrolytes is discussed. Finally, some important issues concerning the fabrication and application are emphasized, which should be paid attention in future.

## Graphical abstract

In this review, the universal strategies on downsizing MOFs and their mechanisms, including reaction parameter‐assisted regulation, coordination adjustment, and other methods are reviewed. Recent progress on NMOFs for battery applications including LIBs, LSBs, Li‐O_2_ batteries is also summed up, in which NMOFs have served as electrodes, separators, and electrolytes.

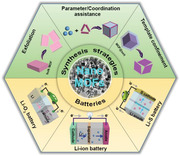

## 1 Introduction

Metal‐organic frameworks (MOFs) have been investigated in many fields,^[^ ^1^ ^]^ and many MOFs with diverse structures have been synthesized and characterized.^[^ ^2^, ^3^, ^4^, ^5^, ^6^, ^7^, ^8^ ^]^ Because of their well‐organized structures, enormous surface areas and pore volumes, well‐defined pore size distributions, and easily‐decorated channels, MOFs have become promising candidates in many applications, including gas storage and separation,^[^ ^9^, ^10^ ^]^ luminescence,^[^ ^11^, ^12^, ^13^ ^]^ thin films,^[^ ^14^, ^15^ ^]^ proton conduction,^[^ ^16^, ^17^ ^]^ catalysis,^[^ ^18^ ^]^ drug delivery,^[^ ^19^, ^20^ ^]^ and energy storage and conversion.^[^ ^21^, ^22^, ^23^, ^24^, ^25^, ^26^ ^]^ However, MOFs normally form large crystals, which greatly weaken physical and chemical processes on their surfaces and restrict their applications. Synthesizing nanoscale MOFs (NMOFs) by downsizing MOFs to the nanoscale range in at least one dimension has become a fascinating research focus in MOF chemistry.^[^ ^27^ ^]^ NMOFs can be used directly or be efficiently modified with other functional materials to provide nanotechnology applications.

Compared with their bulk counterparts, NMOFs retain their inherent structural and chemical characteristics, while also displaying additional properties, such as shorter diffusion pathways for guest transport and more accessible active sites for surface adsorption and reactions. More importantly, NMOFs can be easily functionalized with particular structures and show great potential for various applications.^[^ ^28^ ^]^ For instance, NMOFs are suitable nanocarriers for sustained drug release due to their diverse structures and compositions and their intrinsic biodegradability.^[^ ^29^ ^]^ These advantages ensure electrolyte penetration and improve the electrochemical performance of NMOFs when used as electrodes in diverse battery applications. Their conductivity can be tuned by combining NMOFs with inorganic components, conductive organic polymers (such as polyaniline and polypyrrole), or conductive substrates (carbon nanotube, graphene, nickel foam, carbon paper, etc.).^[^ ^30^, ^31^, ^32^, ^33^, ^34^ ^]^

Many novel NMOFs have been synthesized using various strategies, which involve tuning reaction parameters and coordination reagents, liquid exfoliation, salt‐template confinement, and so on. In 2018, Minkin et al. reviewed the synthesis of NMOFs and analyzed the formation of MOF nanoparticles and the influence of the nucleation mechanism.^[^ ^28^ ^]^ In this review, we summarize recent developments in the main strategies used to form NMOFs and compare their mechanisms. Recent progress in the use of NMOFs in hot battery systems is also highlighted, and some major challenges and perspectives on the development of NMOFs are provided.

## 2 Synthesis Strategies of Nanoscale Metal‐Organic Frameworks

**Figure 1:**
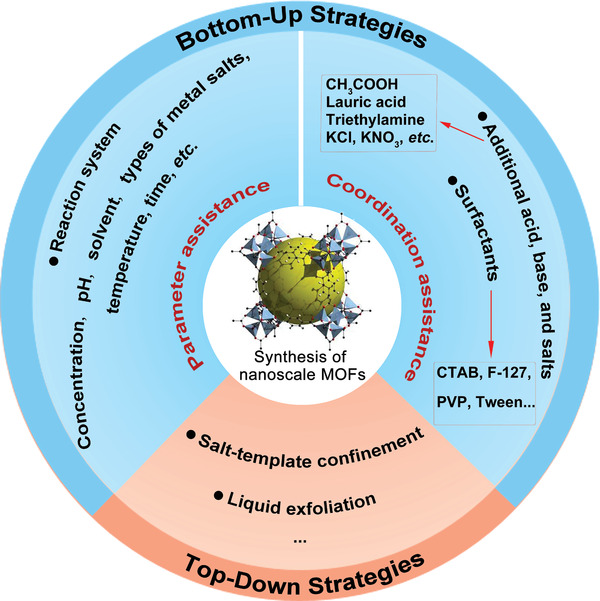
Summary of the synthesis techniques toward NMOFs.

MOFs are commonly constructed by the orderly integration of metal ions/clusters and organic ligands via coordination interactions. Multiple factors influence the formation of MOFs, including metal ions, organic linkers, solvents, template reagents, and reaction conditions, which provide many opportunities for precisely tuning the crystal sizes of target products. Consequently, many different methods have been developed to downsize MOFs to the nanoscale to develop more properties for NMOFs compared with their bulk counterparts. The methods for constructing NMOFs are commonly classified into two major categories, bottom‐up and top‐down strategies (**Figure** **1**). For bottom‐up techniques, two methods are often used to downsize MOFs: 1) Adjusting the reaction parameters (concentration of metal ion and ligands and their ratio, pH, solvent, types of metal salts, temperature, time); 2) introducing coordination reagents (such as acids, bases, inorganic salts, and surfactants). For top‐down techniques, nanoscale structures are obtained by etching techniques such as liquid exfoliation, salt‐template confinement, etc. This section will summarize these two techniques in terms of the method, formation mechanism, and recent progress.

### 2.1 Bottom‐Up Strategies

Similar to the self‐assembly of MOFs, bottom‐up strategies generally begin from well‐fabricated metal ions/clusters and organic linkers due to the inherent tailorability of MOFs. These strategies are similar to self‐assembly, which integrates small atomic or molecular components to construct more complex nanoscale assemblies or directed self‐assemblies based on complex mechanisms and technologies.^[^ ^35^, ^36^ ^]^ The key point of this methodology is to regulate the nucleation rates of different crystal facets by selectively confining growth along the horizontal or vertical direction. Here, bottom‐up strategies are summarized in terms of their reaction parameters and coordination regulations.

#### 2.1.1 Reaction Parameter‐Assisted Synthesis

According to classical nucleation theories, the nucleation and growth of nanomaterials can be divided into three main stages: 1) Nucleation of targeted material precursor under supersaturation; 2) heterogeneous surface reactions of nuclei; 3) continuous enlargement of existing individual nanomaterials. For MOFs, the nucleation and growth rate of crystals is a complex process that is competitively controlled by thermodynamics and kinetics factors. These can be adjusted by varying the concentration or molar ratio of metal ions and organic ligands, solvent, pH, temperature, and so on. Thus, careful attention should be paid to regulating these factors when attempting to downsize MOFs.

**Figure 2:**
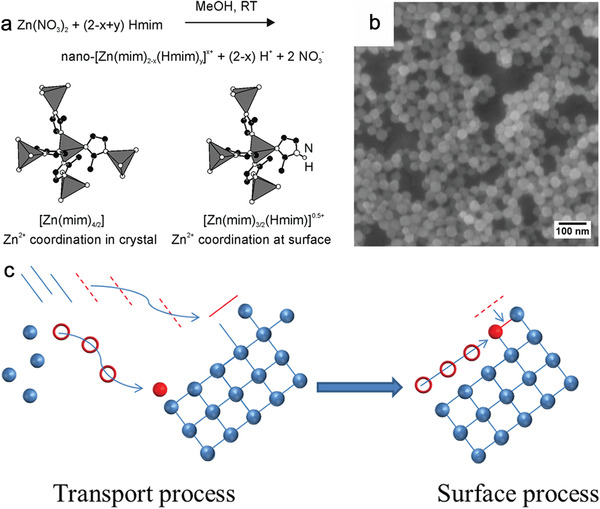
a) Schema illustration of the synthesis of ZIF‐8 nanocrystals capped with neutral Hmim. b) SEM image of the as‐formed ZIF‐8 nanocrystals. Reproduced with permission.^[^ ^40^ ^]^ Copyright 2009, American Chemical Society. c) Schema illustration of the transport and surface processes of MOF growth. Reproduced with permission.^[^ ^41^ ^]^ Copyright 2015, American Chemical Society.

Increasing the ligand concentration provides more nucleation sites and tends to form MOFs dominated by thermodynamic equilibrium,^[^ ^37^, ^38^, ^39^ ^]^ which greatly decreases the size of MOF particles. Using classical zeolitic imidazolate frameworks (ZIFs) as examples, Wiebcke et al. reported the first synthesis of a nanoscale ZIF, in which ZIF‐8 with a smaller size was produced at a higher molar ratio of 2‐methylimidazole (Hmim) to zinc salt, as shown in **Figure** **2**a,b.^[^ ^40^ ^]^ The Hmim itself acts as both a deprotonated linker and a neutral unit to terminate crystal growth and stabilize the positively‐charged nanocrystals (*ζ* potential of 55 mV), leading to the formation of smaller ZIF‐8. Subsequently, Gianneschi et al. explored the growth of ZIF‐8 through in situ liquid cell transmission electron microscopy, which revealed two continuous transportation and surface processes that occurred during ZIF‐8 growth. The former involved the diffusion of both metal and ligand to the nucleus, while the latter involved the movement of both metal and ligand to edge sites or high‐energy sites followed by metal‐ligand coordination (Figure 2c).^[^ ^41^ ^]^

The reaction temperature, time, and solvent also impact the crystal size of MOFs. Decreasing the reaction temperature and shortening the reaction time mainly influence surface reactions and Fickian diffusion of the precursor,^[^ ^42^ ^]^ which suppresses the kinetic growth rate and consequently produces MOFs with smaller crystals. Changing the solvent mixture ratio can change the solution supersaturation, allowing the size of MOF crystals to be adjusted. For instance, Volkmer et al. systematically explored the effects of various synthesis conditions and revealed the significant influence of reaction time, temperature, and solvent on the nucleation process and crystal size.^[^ ^43^ ^]^ By tuning these parameters, the crystal sizes could be adjusted over a broad range, from 40 nm globular particles to 5 µm cubic crystals with narrow size distributions. Li et al. also found that increasing the N,N'‐dimethylformamide (DMF) content in a mixed DMF/H_2_O system reduced the size of Co‐MOF‐74 from 1400 to 20 nm.^[^ ^44^ ^]^ Controlling the pH of the reaction system may also influence the crystallization kinetic but it does not produce smaller particles;^[^ ^45^ ^]^ thus, it is particularly difficult to accurately control nucleation and growth. The influence of metal salts on the crystal sizes of MOFs depends mainly on the reaction activities of salts. Schneider et al. regulated the ZIF‐8 size by changing the zinc salt species and found that ZnBr_2_ produced the microcrystal due to its low activity, while Zn(acac)_2_ (acac = acetylacetonate) produced the smallest crystal size of ≈85 nm.^[^ ^46^ ^]^

Besides the factors mentioned above, the activation methods of reactions such as hydrothermal/solvothermal reactions, microwave irradiation, ultrasound, etc., also play a key role in downsizing MOFs. Only microwave and ultrasonication strategies have been able to produce NMOFs.^[^ ^47^ ^]^ In the case of microwave‐assisted synthesis, the quick thermal conversion of high‐energy microwaves significantly promotes the rapid and efficient heating of reaction solutions, which contributes to fast nucleation. By comparison, ultrasound‐assisted synthesis mainly utilizes the cavitational collapse of vacuum bubbles generated during sonochemical reactions, which can cause fast and intense local heating and high pressures, which rapidly increase the reaction kinetics. These two strategies provide a general method for the design and construction of NMOFs, such as MIL‐101‐NH_2_,^[^ ^48^ ^]^ MIL‐88A,^[^ ^45^ ^]^ Zn_3_(BTC)_2_,^[^ ^49^ ^]^ HKUST‐1,^[^ ^50^ ^]^ [Tb(BTC)(H_2_O)_6_]_m_,^[^ ^51^ ^]^ etc. Hydrothermal/solvothermal methods have also been selected to directly obtain NMOFs by using adding additives or adjusting the solvent ratio. Li et al. reported a series of Co‐MOF‐74 crystals with diameters decreasing from 1400 nm (rods) to 20 nm (nanofibers) via a simple solvothermal strategy.^[^ ^44^ ^]^ Ruan et al. also used a hydrothermal method to directly synthesize nanozised NH_2_‐MIL‐125 (Ti) with a particle size of 300 nm, high phase purity, and physicochemical properties.^[^ ^52^ ^]^

**Table** **1** lists representative examples of NMOFs obtained from these above‐mentioned strategies in terms of their preparation method, particle size, and morphology. For hydrothermal/solvothermal techniques, it is a simple and scalable method for the synthesis of NMOFs in high yield and low cost. The temperature, time, rate, and pressure are precisely controlled to regulate the crystal size in all size regimes. However, a closed system could make it quite difficult to observe the occurred reactions and their mechanisms, and high temperature could result in substantial energy consumption. The sensitivity to the experimental conditions also makes it complicated to accurately control the NMOF structure in different batches and laboratories. As to microwave/ultrasound irradiation techniques, it shows a fast generation of NMOFs with smaller particle sizes by controlling radiation time, but suffers from local overheating, slow cooling process, and more by‐products. By comparison, solution‐based direct precipitation offers a broader size distribution, faster kinetics, lower reaction temperatures, safer reaction processes, and higher yields.

**Table 1:** Summary of some classical NMOFs materials in the literature

Entry	Compound	Morphology	Size [nm]	Synthesis method	Ref.
1	IR‐MOF‐n	Regular or truncated octahedral	200 to 300 nm	Surfactant‐assisted method	^[^ ^73^ ^]^
2	MOF‐5(Zn)	Nanoparticles	100 to 200 nm	Surfactant and/or capping groups assisted solvothermal method	^[^ ^139^ ^]^
3	MOF‐5(Zn)	Nanoparticles	30 to 150 nm	Direct precipitation in solution	^[^ ^140^ ^]^
4	MOF‐5(Zn)	Nanoparticles	40 nm	Direct precipitation in solution	^[^ ^141^ ^]^
5	Fe‐soc‐MOF	Cubic to spherical	1 µm to 200 nm	Tween‐85 regulated reflux method	^[^ ^76^ ^]^
6	Fe‐MIL‐88‐NH_2_	Bipyramidal hexagonal prism	Length: 50 ± 5 nm Width: 30 ± 5 nm	Non‐ionic triblock copolymer and acetic acid regulated in situ growth method	^[^ ^142^ ^]^
7	Fe‐MIL‐88‐NH_2_	Octahedral	≈200 nm	Acetic regulated in situ growth	^[^ ^143^ ^]^
8	MIL‐101(Cr)	Nanoparticles	50 nm	Microwave irradiation technique	^[^ ^144^ ^]^
9	MIL‐53(Al)	Nanoparticles	20 to 60 nm	Solvothermal method	^[^ ^145^ ^]^
10	NH_2_‐MIL‐125 (Ti)	Nanocubes	≈300 nm	Solvothermal method	^[^ ^52^ ^]^
11	Cu(bdc)(S) MOF	Square	300*300*30 to 50*50*20 nm	Acetic acid regulated growth	^[^ ^59^ ^]^
12	HKUST‐1(Cu)	Cubic	20 to 50 nm	Acetic acid or dodecanoic acid regulated in situ growth	^[^ ^61^ ^]^
13	HKUST‐1(Cu)	Octahedral	400 to 90 nm	Dodecanic acid or benzoic acid regulated in situ growth	^[^ ^63^ ^]^
14	Cu‐imidazolate MOF	Ellipsoid‐like shape	Length: 150 to 500 nm width: 0.25 to 1 µm	NH_3_⋅H_2_O regulated in situ growth	^[^ ^64^ ^]^
15	HKUST‐1(Cu)	Cube to octahedron	300 to 500 nm	CTAB regulated in situ growth	^[^ ^72^ ^]^
16	HKUST‐1(Cu)	Nanoparticles	Sub 5 nm	Metal‐organic‐gel route	^[^ ^146^ ^]^
17	HKUST‐1(Cu)	Nano‐cubes Nano‐flakes	50–200 nm 15–25 nm	Two‐ligand regulation strategy and self‐assembly	^[^ ^147^ ^]^
18	HKUST‐1(Cu)	Nanoparticles	An average of 25 nm	CO_2_ pressure regulated ion liquid method	^[^ ^148^ ^]^
19	HKUST‐1(Cu)	Nanooctahedrons	≈280 nm	POM regulated microwave irradiation	^[^ ^149^ ^]^
20	Cu(ndc)_2_(dabco)	Nanocube to nanorod	80 ± 20 nm	Acetic acid and pyridine regulated solvothermal reaction	^[^ ^150^ ^]^
21	ZIF‐8(Zn)	Rhombic dodecahedron	20 to 46 nm	In situ growth	^[^ ^40^ ^]^
22	MAF‐4 (Zn)	Cube to particle	1 µm to 40 nm	Synthesis parameters regulated solvothermal reaction	^[^ ^44^ ^]^
23	ZIF‐8(Zn)	Nanoparticles	78 to 27 nm	Temperature regulated in situ growth	^[^ ^66^ ^]^
24	ZIF‐7(Zn)	Nanoparticles	30 nm	Direct precipitation in solution	^[^ ^151^ ^]^
25	ZIF‐8(Zn)	Rhombic dodecahedral particles	178 ± 8 to 227 ± 10 nm	Surfactant‐assisted static growth	^[^ ^152^ ^]^
26	ZIF‐8(Zn)	Rhombic dodecahedra	≈60 nm	In situ growth	^[^ ^153^ ^]^
27	ZIF‐8(Zn)	Truncated nanocubes	≈110 nm	CTAB modified hydrothermal method	^[^ ^154^ ^]^
28	ZIF‐8(Zn)	From cube to rhombic dodecahedron	50 nm to 2 µm	Microreactor confined synthesis	^[^ ^155^ ^]^
29	ZIF‐11(Zn)	Nanoparticles	36 ± 6 nm	Centrifugation synthesis	^[^ ^156^ ^]^
30	ZIF‐67(Co)	Nanosheets	4.5 nm thickness	Bottom‐up strategy with salt‐template confinement	^[^ ^78^ ^]^
31	ZIF‐67(Co)	Nanocubes	≈730 nm	CTAB‐mediated method	^[^ ^157^ ^]^
32	ZIF‐67(Co)	Truncated nanocubes	≈150 nm	Surfactant‐assisted solvothermal method	^[^ ^158^ ^]^
33	ZIF‐67(Co)	Polyhedral	50 ± 10 nm	Direct precipitation in solution	^[^ ^68^ ^]^
34	ZIF‐67(Co)	Polyhedral	75 to 385 nm	Hydrothermal method	^[^ ^159^ ^]^
35	UiO‐66(Zr)	Octahedral particles	500 nm to 2 µm	Acetic acid and TEA regulated precipitation	^[^ ^69^ ^]^
36	UiO‐66(Zr)	Octahedral particles	340 ± 30 nm	Solvothermal method	^[^ ^152^ ^]^
37	UiO‐66(Zr)	Nanoparticles	10 to 125 nm	Stock solution aging followed by solvothermal method	^[^ ^160^ ^]^
38	Zr‐MOF‐NH_4_	Nanoparticles	≈17 to 63 nm	Solvothermal method	^[^ ^161^ ^]^
39	Co‐MOF‐74	Nanofibers	20 nm	Solvothermal method with controlled solvent ratios	^[^ ^43^ ^]^
40	Dy(BTC)H_2_O	Rod‐like to spherical	3 µm to 71 nm	Capping reagents regulated in situ growth	^[^ ^57^ ^]^
41	Ca‐SDB MOF	—	400–600 nm to 700 nm	NH_3_⋅H_2_O and lauric acid regulated solvothermal reaction	^[^ ^60^ ^]^
42	Prussian blue	Nanocubes	≈170 nm	Trisodium citrate regulated in situ growth	^[^ ^71^ ^]^
43	Prussian blue	Nanocubes Nanospheres	≈90 nm ≈80 nm	Surfactant‐assisted solvothermal method	^[^ ^162^ ^]^
44	Prussian blue	Nanocubes	≈200 nm	Direct precipitation in solution	^[^ ^163^ ^]^
45	Prussian blue analogue	Nanocubes	Length: 60 ± 10 nm Height: 20 ± 5 nm	Microemulsion technique	^[^ ^164^ ^]^

#### 2.1.2 Coordination‐Assisted Synthesis

**Figure 3:**
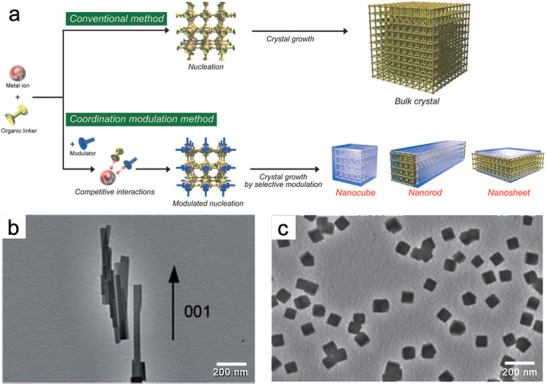
a) Coordination regulation strategy for the fabrication of NMOFs. b,c) TEM images of MOF nanocrystals with different morphologies obtained by coordination reagent regulation. Reproduced with permission.^[^ ^56^ ^]^ Copyright 2009, Wiley‐VCH.

Coordination reagents—sometimes termed as capping reagents—have been used to restrict crystal growth and control the morphologies of metal and semiconductor nanoparticles by reacting with particle surfaces to inhibit further molecular addition from the mother liquor.^[^ ^53^, ^54^, ^55^ ^]^ Motivated by this, capping reagents have been introduced to regulate the sizes and morphologies of MOFs via competitive coordination with ligands or metal ions/nodes or linkers. This prevents coordination interactions between metal ions and linkers to form NMOFs (**Figure** **3**a).^[^ ^56^ ^]^ Furthermore, the selective coordination modulation can change the rate of framework extensions in different directions, resulting in controllable morphologies of MOF nanocrystals (Figure 3b,c). This section summarizes recent advances in the coordination‐assisted fabrication of NMOFs, mainly focusing on the regulation of additional acids, bases, inorganic salts, and surfactants. The corresponding reaction mechanisms are also analyzed.

##### Additional Acids, Bases, and Salts

Linkers with single functional groups are often used as additives to terminate crystal extension by blocking binding sites, thereby decreasing the crystal sizes. The regulation mechanism requires balancing the competition between the kinetic nucleation of target MOFs and the coordination effect of linkers, which leads to the formation of MOFs with a small size. The introduced acids or bases modify the acid‐base environment of the reaction system, but they can also act as chelating agents that coordinate with metal sites. The rapid deprotonation and nucleation at higher pH and the inhibitory effect of capping groups on the growth produce MOFs with small crystals.^[^ ^57^ ^]^ Small‐molecule acids are commonly used as capping reagents, including hydrofluoric acid (HF),^[^ ^58^ ^]^ acetic acid,^[^ ^59^ ^]^ lauric acid,^[^ ^60^, ^61^ ^]^ benzoic acid,^[^ ^62^ ^]^ salicylic acid,^[^ ^43^ ^]^ and fumaric acid.^[^ ^63^ ^]^ As a representative example, Guo et al. reported the facile synthesis of size‐controlled Zr‐MOF, Zr‐1,4‐benzenedicarboxylate(UiO‐66) crystals by adding HF. The results revealed that the strongly electronegative fluorine ion bonded to zirconium in the secondary building unit, which balanced the charges of the framework caused by the missing linker. It also competed with the ligands for zirconium coordination sites, thereby controlling the nucleation and growth of UiO‐66 crystals.^[^ ^58^ ^]^ The presence of HF also enhanced thermal stability and porosity.

Notably, different acids display different influences on crystal size. Wang et al. showed that Cu_3_(BTC)_2_ (BTC = benzene‐1,3,5‐tricarboxylate) produced 90 nm spherical crystals and 400 nm octahedral crystals in the presence of monocarboxylic dodecanoic acid and benzoic acid, respectively.^[^ ^62^ ^]^ However, microsized Cu_3_(BTC)_2_ crystals were obtained in the absence of an acid mediator. This may be ascribed to the role of acids, which acted as nucleation inhibitors that changed the coordination equilibrium of the crystal surface, or competed with BTC ligand to prevent the crystal growth. Additionally, a high concentration of benzoic acid slowed the nucleation of the Cu_3_(BTC)_2_ framework, which produced larger crystals with greater size polydispersities.^[^ ^61^ ^]^ Similarly, Pang et al. compared the regulatory effect of acetic acid, lauric acid, benzoic, and fumaric acid on the sizes and morphologies of In‐NDC‐MOF (NDC = 1,4‐naphthalenedicarboxylate) particles. They found that only acetic acid induced the formation of MOFs with small crystals of about 6 µm in length and 1 µm in width.^[^ ^63^ ^]^ Although much progress has been achieved for using acid to regulate MOF crystal sizes, acid additives tend to promote crystal growth because they decrease the nucleation rate due to competitive coordination with ligands; thus, there is a need for alternatives methods to downsize MOFs.

**Figure 4:**
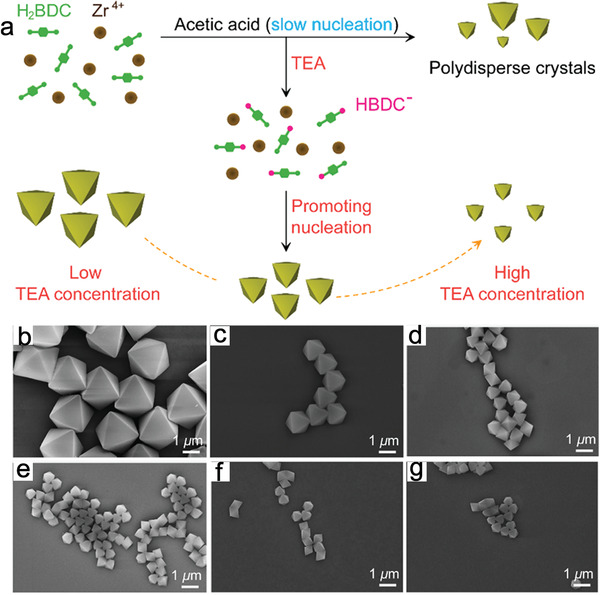
a) Schematic illustration of the acid and base coregulated synthesis of UiO‐66 crystals. b–g) SEM images of UiO crystals obtained under different TEA concentrations. Reproduced with permission.^[^ ^69^ ^]^ Copyright 2017, American Chemical Society.

Compared with acids, bases are more suitable for tuning the formation of NMOFs. Bases promote crystal nucleation by forming many tiny crystal seeds through deprotonation, and they also act as structure‐directing agents to promote the formation of MOF crystals. Several bases have been used to fabricate NMOFs, such as NH_3_⋅H_2_O,^[^ ^64^ ^]^ triethylamine (TEA),^[^ ^65^ ^]^ and *n*‐butylamine.^[^ ^66^ ^]^ Li et al. used TEA as a regulating reagent to reduce the particle size of ZIF‐8 down to ≈15 nm.^[^ ^67^ ^]^ Hu et al. also downsized ZIF‐67 to 50 ± 10 nm by using a similar strategy.^[^ ^68^ ^]^ When NH_3_·H_2_O was used as an additive, the ligand was deprotonated, and it coordinated with metal ions or noded to form a coordination complex. When used in low concentrations, NH_3_·H_2_O accelerated ligand exchange and formed crystal seeds, which produced small MOFs. However, a high concentration of NH_3_·H_2_O prevented the formation of many crystal seeds and suppressed ligand exchange, which produced larger MOFs.^[^ ^64^ ^]^ Interestingly, acids and bases can be used together to adjust the crystal sizes and morphologies of MOFs in some cases, but they play different roles. Lu et al. used acid and base coregulation to synthesize UiO‐66 with tunable morphologies and large sizes (**Figure** **4**a),^[^ ^69^ ^]^ in which acetic acid was used to tune the morphology and TEA decreased the crystal size of UiO‐66. As the TEA concentration increased from 0 to 8 mm, the average crystal size of UiO‐66 crystals decreased from 2040 to 512 nm (Figure 4b–g). TEA promoted the rapid formation of HBDC^−^, which accelerated the nucleation process and formed monodisperse crystals with tunable sizes.

**Figure 5:**
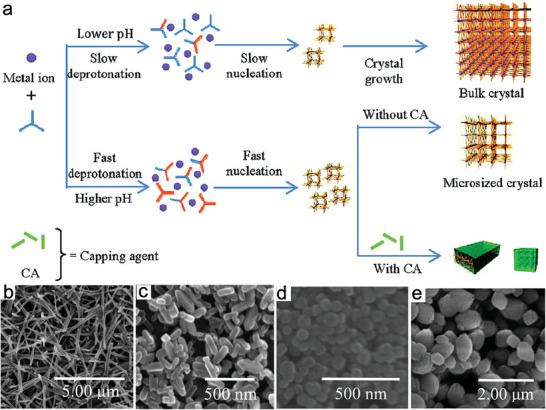
a)The regulation mechanism of additional acids or bases on the formation of NMOFs. b–e) SEM images of Dy(BTC)H_2_O obtained at different amounts of sodium acetate. Reproduced with permission.^[^ ^57^ ^]^ Copyright 2012, American Chemical Society.

Besides acids/bases regulation, inorganic salts can also significantly influence the size of MOFs via one of two main regulatory mechanisms. In the first, inorganic salt acts as an acid or base to adjust the pH of the synthesis solution, and then they change the coordination concentration and rate between metal ions and organic linkers, thereby downsizing MOFs. The second mechanism uses the introduced inorganic anions to competitively complex with metal ions, which controls the crystallization rate of the MOF. For example, Zhang et al. used sodium acetate (NaAc) as a capping agent to downsize Dy(BTC)H_2_O MOF from rod‐like microcrystals (3 µm) to spherical nanocrystals (71 nm) by changing the concentration ratio of NaAc to BTC (**Figure** **5**a–e).^[^ ^57^ ^]^ A higher NaAc concentration increased the pH of the synthesis solution, and then deprotonated BTC ions coordinated with metal ions, which increasingly accelerated the nucleation rates and decreased the crystal size and anisotropy. Sun et al. added several inorganic salts (NaNO_3_, NaCl, NaBr, KCl, and KBr) to the reaction system to tune the size of HKUST‐1 crystals.^[^ ^70^ ^]^ It was revealed that sodium ions were adsorbed on the surfaces of ligands through electrostatic interactions, which prohibited the formation of crystal growth units. When increasing sodium ion concentration, the supersaturation of crystal growth units was further suppressed, which increased the crystal size. Potassium ions showed a similar effect on regulating the MOF size, probably due to greater suppression because of the larger ionic radius. No obvious influence on MOF size was observed for the counter anions. Metal cations can also affect the surface energies of crystal faces, and this can be exploited to tune the morphologies of NMOFs. Trisodium citrate has also been used as an additive to alter the crystal sizes of MOFs by reacting trisodium‐citrate‐coordinated metal ions with ligand‐metallic ion complexes, thereby controlling slow crystallization to generate NMOFs.^[^ ^71^ ^]^

##### Surfactants

**Figure 6:**
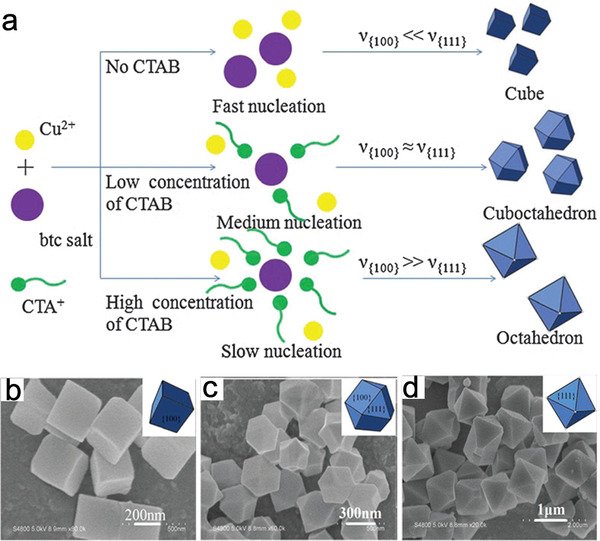
a) The proposed schematic illustration of the regulation on the formation of HKUST‐1 by surfactants. b–d) SEM images of HKUST‐1 obtained by changing the concentration of CTAB. Reproduced with permission.^[^ ^72^ ^]^ Copyright 2012, The Royal Society of Chemistry.

Surfactants regulate the sizes and morphologies of the final MOFs via chemical interactions with metal ion sites or organic ligands. These interactions change the free energies of different growing facets and slow their relative growth rates, thereby promoting the formation of NMOFs. Currently, two kinds of surfactants are used to regulate MOF sizes: 1) Cationic surfactants represented by cetyltrimethylammonium bromide (CTAB), which has a positively‐charged head and a long hydrophobic tail; 2) non‐ionic surfactants, including polyvinylpyrrolidone, P123 (EO_20_PO_70_EO_20_), F127 (EO_97_PO_69_EO_97_), and Tween‐85, which are inexpensive, nontoxic, and biodegradable. When CTAB was introduced into a reaction system, dissociated CTA^+^ combined with ligands via electrostatic interactions, while its hydrophobic tail prevented metal ions or nodes from coordinating with ligands, which decreased the nucleation rate. In this way, however, MOFs with large crystal sizes were obtained. Sun et al. used CTAB to regulate the morphology and size of HKUST‐1.^[^ ^72^ ^]^ As the CTAB concentration increased, the MOF size increased from 300 to 1000 nm, while the morphology changed from cubic to octahedral (**Figure** **6**a–d). CTAB showed a similar effect on the size of MOF‐5 and other MOFs.^[^ ^73^, ^74^ ^]^ By comparison, non‐ionic surfactants form micelles and electrostatically interact with metal ions or nodes to alter the formation process of MOF nanocrystals, thereby decreasing the crystal sizes of MOFs. Chen and coworkers introduced non‐ionic triblock copolymer surfactants P123 and F127 to realize the morphology and size regulations of ZIF‐8.^[^ ^75^ ^]^ Bu et al. obtained a series of Fe‐soc‐MOF nanocrystals with different sizes from 1 µm to 200 nm by adjusting the Tween‐85 content.^[^ ^76^ ^]^

In summary, bottom‐up strategies, including the addition of acid, base, and surfactant, and the adjustment of synthesis parameters, have been proven to be valuable at restricting the crystal sizes of NMOFs.

### 2.2 Top‐Down Strategies

**Figure 7:**
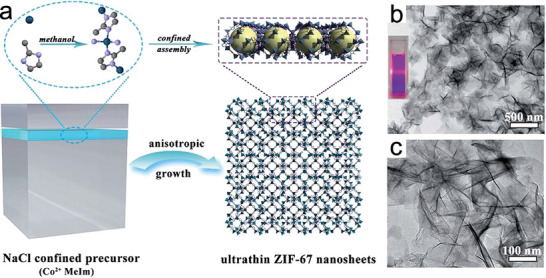
a) Schematic illustration for the salt template confinement process of ZIF‐67 nanosheets. b,c) TEM images of the as‐synthesized ZIF‐67 nanosheets. Reproduced with permission.^[^ ^79^ ^]^ Copyright 2017, The Royal Society of Chemistry.

Top‐down strategies, such as liquid exfoliation and salt‐template confinement, have also been used to prepare NMOFs by disintegrating the bulky MOFs into small fragments, especially in the regulation of 2D MOF nanosheets. Zhang et al. synthesized a series of 2D MOF nanosheets (M‐TCPP, M = Zn, Cu, Cd, or Co, TCPP = tetrakis(4‐carboxyphenyl)porphyrin)) by exfoliation technique.^[^ ^77^ ^]^ By introducing 4,4’‐dipyridyl disulfide as an intercalating reagent, Zhou et al. exfoliated layered MOF crystals into ultrathin 2D MOF nanosheets (≈1 nm).^[^ ^78^ ^]^ The exfoliation of layed MOFs into nanosheets typically requires capping reagents. It is also worth noting that the exfoliation method often results in low yield and are only suitable for those MOFs whose bulk crystals are layed compounds, therefore having certain limitations. Recently, Dong et al. pioneered the development of a salt‐template confinement technique to fabricate high‐quality and ultrathin zeolite imidazole framework (ZIF‐67) nanosheets (**Figure** **7**).^[^ ^79^ ^]^ The obtained ZIF‐67 nanosheets possessed an average thickness of 4.5 nm, which corresponds to three structural coordination layers. During preparation, the volume of solvent and the molar ratio of precursor‐to‐salt played key roles in controlling the morphology of ZIF‐67 nanosheets. The limited volume of methanol solvent in the gaps of the NaCl template confined the growth space and direction of ZIF‐67, while the low precursor ratio guaranteed the orientated growth of ZIF‐67 along the NaCl microcrystal plane to form ultrathin 2D ZIF‐67 nanosheets. This effective salt‐template assisted strategy was low‐cost and displayed easy recoverability, and could be extended to the synthesis of other 2D MOF nanomaterials.

## 3 Application of Nanoscale Metal‐Organic Frameworks in Batteries

In today's society, the explosive development of portable electronic devices and hybrid electric vehicles has driven the pursuit of renewable electrical energy storage devices with greater energy and power densities. Among the various emerging energy storage systems, batteries are the primary technology, but they suffer from inferior power densities, which requires the extensive exploration of new materials with higher electrochemical performances.^[^ ^24^ ^]^ MOFs stand out from the many other functional materials and exhibit great potential in battery applications. Compared with pristine bulk MOFs, NMOFs are more suitable for use as battery electrodes because: 1) Redox‐active metal centers and/or the defects in organic linkers enable multi‐electrons reactions and promote charge transfer inside the frameworks; 2) their nanoscale sizes provide closer contact with the electrolyte for faster ion transfer; 3) the regular channels and pores are beneficial for electrolyte permeation, the accommodation of active substances/reaction products, and buffering volume changes during charging–discharging processes; 4) the high surface area provides large electrode/electrolyte interface, decreases the local current density, and reduces Li dendrite formation.^[^ ^80^ ^]^ This section summarizes the recent application progress of NMOFs used in batteries, including lithium‐based batteries, sodium‐based batteries, and separator and electrolyte systems.

### 3.1 Lithium‐Based Batteries

Lithium‐based batteries, which primarily include lithium‐ion batteries (LIBs), lithium‐sulfur batteries (LSBs) and lithium‐oxygen (Li‐O_2_) batteries, have been widely studied in recent years. These systems are generally comprised of a cathode, anode, separator, and electrolyte, all of which are closely associated with battery performance. Electrode materials determine the capacity, cycling life, power and energy densities, while the separator and electrolyte play critical roles in homogeneous Li^+^ ion transfer. Porous NMOFs with tunable structures are promising materials and the smaller crystal size of NMOFs can decrease the Li^+^ transport distances and promote the insertion/extraction of Li^+^.^[^ ^81^, ^82^ ^]^ Considering the dominant position of nanotechnology for future energy needs, NMOFs are more suitable for use in lithium‐based batteries than their bulk counterparts. Typical examples of NMOFs in batteries are listed in **Table** **2**.

**Table 2:** Nanosized MOFs for batteries

Lithium‐ion batteries							
Sample	PS	Active mass	CR/CN	Initial CE	TC/ICC/IDC	RC/rate	Ref.
Asp‐Cu	100–200	—	98.5%/200 (0.05 C)	26.6%	275/—/—	—/400	^[^ ^89^ ^]^
Co‐COP	—	1.5 mg cm^−2^	593/1000 (1 C)	68.3%	—/1107/1620	154/10 C	^[^ ^90^ ^]^
Co‐BDCN	45–55	1.0 mg cm^−2^	1132/100 (0.1 C)	70.54%	—/1015/1439	538/2 C	^[^ ^91^ ^]^
Mn‐UMOFN	2–7 thickness	1.2–1.5 mg cm^−2^	1187/100 (0.1 C)	57%	1392/—/—	701/2 C	^[^ ^92^ ^]^
u‐CoTDA	1–10 thickness	—	790/400 (1 C)	75.2%	—/1226/1631	694/2 C	^[^ ^93^ ^]^
Sn‐PMA	557 thickness	2.6 mg cm^−2^	707/400 (0.8 C)	42.2%	—/661/1567	226/1.6 C	^[^ ^94^ ^]^
UiO‐66	38	0.7–1.3 mg	65%/100 (0.2 C)	—	194/—/—	—/5 C	^[^ ^96^ ^]^
F‐MOF‐3	—	—	620/500 (0.5 C)	—	—	548/1 C	^[^ ^97^ ^]^
HP‐NENU‐5/CC	—	1.8–2.2 mg cm^−2^	1072/400 (1 C)	68.1%	—/1595/2346	662/2 C	^[^ ^98^ ^]^
PMo_10_V_2_‐ILs@MIL‐100	300	1 mg	600/400 (1 C)	66.94%	—/1666/1115	347.5/3 C	^[^ ^99^ ^]^
BP/NiCo‐MOF	6 thickness	1.5 mg cm^−2^	569/250 (2 C)	60.89%	—/1512/2483	398/5 C	^[^ ^100^ ^]^
POMOF/rGO	200 nm	—	1075/100 (0.05 C)	64.1%	—/—/—	365/3 C	^[^ ^101^ ^]^
SnO_x_@UiO‐66	50 nm	—	994/100 (0.05 C)	53%	—/—/2590	358/2 C	^[^ ^102^ ^]^
Cu_3_(HHTP)_2_	20–40 nm	—	95/100 (0.095 C)	–	95.61/—/105	85/0.19 C	^[^ ^104^ ^]^

Lithium‐sulfur batteries							
Sample	PS	S content	CR/CN	SSA	ICC/IDC	RC/rate	Ref.
S/ZIF‐8	100–200	≈50 wt%	553/300 (0.5 C)	—	—/—	710/1 C	^[^ ^107^ ^]^
MIL‐100(Cr)/S@							
155 + 50% C	—	48 wt%	—/60 (0.1 C)	360 (MOF)	—/1580	—/—	^[^ ^108^ ^]^
S@ZIF‐8	—	30 wt%	510/100 (0.1 C)	—	—	450/1 C	^[^ ^109^ ^]^
S@Cu‐TDPAT	100 nm	50 wt%	745/500 (1.674 C)	1473 (MOF)	—/995	523/8.37 C	^[^ ^111^ ^]^
	200 nm	53 wt%	631/300 (0.837 C)	1532 (MOF)	—/—	—/—	
	500 nm	53.4 wt%	436/300 (0.837 C)	1618 (MOF)	—/—	—/—	
CNT@UiO‐66‐S	50–200 nm	≈60 wt%	80.2%/900 (3.35 C)	14.01	—/1230	608/1.675 C	^[^ ^112^ ^]^
ZIF‐67‐5‐S	—	75 wt%	521.1/550 (0.5 C)	369 (MOF)	—/—	510/1.6 C	^[^ ^113^ ^]^
PPyNTs@UIO‐66‐S	≈125 nm	75.7 wt%	996/200 (0.1675 C)	1168.3 (MOF)	—/1215	808/3.35 C	^[^ ^114^ ^]^
ZIF‐67‐S‐PPy	—	60 wt%	354/200 (0.1675 C)	21.44	—/1092.5	—/—	^[^ ^115^ ^]^

Lithium‐oxygen batteries							
Sample	PS	Morphology	DV/CV	DC/rate	CN	LC/rate	Ref.
Co‐MOF‐74	1400	Rod‐shape	2.67/—	4710/100	—		^[^ ^43^ ^]^
	800	Nanorod	2.66/—	4880/100	—		
	20	Nanofiber	2.74/—	11 350/100	8	1000/0.25 C	
Co‐MOF	3.79 thickness	Nanosheet	2.71/4.09	5770/200	16	1000/0.2 C	^[^ ^119^ ^]^
Ni‐MOF	3.62 thickness	Nanosheet	2.73/4.13	5050/200	17	1000/0.2 C	
Mn‐MOF	5.30 thickness	Nanosheet	2.78/4.06	7387/200	110	1000/0.2 C	

PS: Particle size (nm); CR: Capacity retention (mAh g^−1^); CN: Cycling number; CE: Coulombic efficiency; TC: Theoretical capacity (mAh g^−1^); ICC: Initial charging capacity (mAh g^−1^); IDC: Initial discharging capacity (mAh g^−1^); RC: Reversible capacity (mAh g^−1^)/rate (mA g^−1^); SSA: Specific surface area (m^2^ g^−1^); DV: Discharging voltage (V); CV: Charging voltage (V); LC: Limited capacity (mAh g^−1^)/rate (mA g^−1^); 1 C = 1000 mA g^−1^; Abbreviations: Asp: Aspartic acid; BDCN: Terephthalonitrile; H_2_TDA: 2,5‐thiophenedicarboxylic; PMA: 1,2,4,5‐benzene‐tetracarboxylic acid; BP: Black phosphorus; HHTP: 2,3,6,7,10,11‐hexahydroxytriphenylene; H_6_TDPAT: 2,4,6‐tris(3,5‐dicarboxylphenylamino)‐1,3,5‐triazine; Note: In ref. ^[^ ^133^ ^]^, the detailed C value was converted from the theoretical capacity of Cu_3_(HHTP)_2_; the S content in composite was calculated by using TG result.

#### 3.1.1 Lithium‐Ion Batteries

LIBs are promising energy storage systems and have a high theoretical energy density, low self‐discharge, long lifespans, and a lower memory effect. Thus, they have been introduced into commercial markets for portable electronic devices and HEVs.^[^ ^22^ ^]^ In recent years, an important breakthrough has been achieved for applying MOFs in LIBs because their structures provide Li^+^ storage sites and buffer space sites during charge/discharge.^[^ ^83^, ^84^, ^85^, ^86^, ^87^, ^88^ ^]^ However, the poor conductivity of MOFs usually results in a poor capacity for LIBs. Decreasing the particle size of MOFs to shorten ion and electron transfer distances is an alternative strategy to improve the conductivity of NMOFs, making them more ideal for LIB applications than bulk MOFs.

NMOFs are most frequently used as anodes in LIBs. Zhao et al. used a Cu‐based MOF (Asp‐Cu, asp = an amino acid), in a 1D nanorod morphology with a diameter of 100–200 nm, as the anode for LIBs.^[^ ^89^ ^]^ Within the voltage range of 0.01–3.0 V, the specific capacity of the electrode decreased in the first 10 cycles, then increased slightly in the following 200 cycles. The good cyclability was ascribed to volume changes during charge/discharge that completely opened the pores of Asp‐Cu and accelerated the penetration of electrolyte throughout the electrode. The low actual capacities limit their application in LIBs. Later, an anode based on 1D Co‐based nanowires several micrometers in length and 30 nm in diameter were fabricated and delivered a high Li storage capacity of over 1100 mAh g^−1^ at 20 mA g^−1^. This performance was ascribed to intercalation‐like reversible structural deformation and the reduction of Co^2+^ to Co^0^.^[^ ^90^ ^]^ Yang et al. also synthesized a Co‐BDCN MOF (BDCN = terephthalonitrile) nanorod with a diameter range from 45 to 55 nm, which achieved a reversible capacity of 1132 mAh g^−1^ at 100 mA g^−1^ after 100 cycles.^[^ ^91^ ^]^ During discharge‐charge cycles, the electron‐donating effect of the oxygen and nitrogen atoms in the amide groups, as well as the benzene ring, were the main reasons for Li^+^ storage.

**Figure 8:**
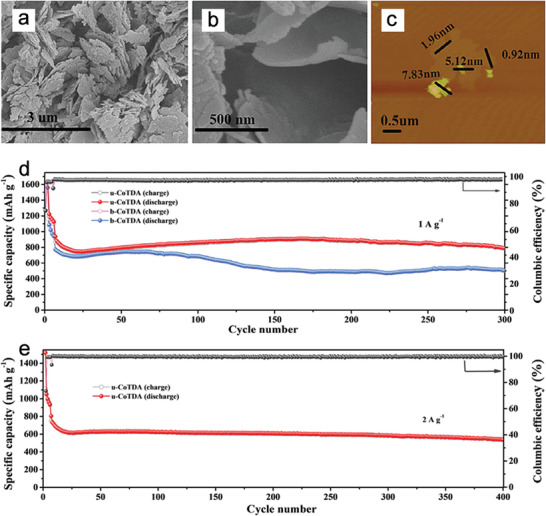
a,b) SEM and c) AFM images of the as‐prepared u‐CoTDA. Cycling performance of u‐CoTDA and b‐CoTDA at current densities of d) 1 A g^−1^ and e) 2 A g^−1^ versus Li^+^/Li. Reproduced with permission.^[^ ^93^ ^]^ Copyright 2017, Wiley‐VCH.

2D NMOFs have also been used as LIB anodes and provided ion diffusion and electron transfer pathways. For instance, Hu et al. reported ultrathin manganese‐based MOF nanosheets (Mn‐UMOFN, thickness range of 1.8–7.0 nm and mostly < 3 nm), where both the metal and ligand possessed redox activities.^[^ ^92^ ^]^ When used as a LIB anode, Mn‐UMOFN exhibited a highly‐reversible capacity (1187 mAh g^−1^ at 100 mA g^−1^ after 100 cycles, theoretical capacity: 1392 mAh g^−1^), excellent rate capability (701 mAh g^−1^ at 2 A g^−1^), rapid Li^+^ diffusion coefficient (2.48 × 10^−9^ cm^2^ s^−1^), and low charge–discharge potential. The synchrotron‐based soft X‐ray spectroscopy (sXAS) results of O K‐edge and Mn L‐edge indicated that both the aromatic chelating ligands and Mn^2+^ centers participated in Li^+^ storage. Similarly, ultrathin cobalt‐based MOF nanosheets (u‐CoTDA) with thicknesses ranging from 1 to 10 nm were prepared and showed a highly‐reversible capacity of 790 mAh g^−1^ at 1.0 A g^−1^after 300 cycles and excellent rate performance when used as a LIB anode (**Figure** **8**).^[^ ^93^ ^]^ Co K‐edge X‐ray absorption near edge structure spectroscopy, O K‐edge sXAS, and EPR techniques revealed that the reduction/oxidation processes of Co centers and organic ligands of u‐CoTDA were the main factors that contributed to the outstanding electrochemical performance. Despite the reversible electrochemical activities between M^2+^ and M^0^ (M = Mn, Co) during discharge‐charge cycles, the structures of Mn‐UMOFN and u‐CoTDA collapsed. Very recently, a layered Sn‐based MOF (Sn‐PMA, PMA = 1,2,4,5‐benzenetetracarboxylic acid) with a thickness of 550 nm was reported and delivered a high capacity of 707 mAh g^−1^ at 800 mA g^−1^ after 400 cycles with a contribution from Li^+^ intercalation pseudocapacitance.^[^ ^94^ ^]^ During lithiation, the Sn‐PMA structure changed from long‐range order to short‐range order due to the breakage of some Sn and O coordination bonds.

Besides the 1D and 2D NMOFs mentioned above, NMOF nanoparticles have also been used as LIB anodes and have shown better electrochemical performance than bulk MOFs. Recently, Hu et al. fabricated nanoscale Basolite F300‐like Fe‐BTC and used it as an anode in LIBs.^[^ ^95^ ^]^ The robust structure, high porosity, active reaction sites of the conjugated organic carboxylates and its nanoscale size (≈200 nm) played key roles that guaranteed its electrochemical performance. As expected, this Fe‐MOF‐based anode delivered high reversible capacity and good rate capacity that were superior to those of bulk Fe‐BTC material. In addition, a nanoscale UiO‐66 without redox‐active metal nodes was also used as a LIB anode.^[^ ^96^ ^]^ Density functional theory calculations indicated that charge transfer in the non‐redox UiO‐66 framework during lithiation occurred from Li to the node oxygen and carboxylate oxygen. That is, it involved anions rather than cations or aromatic rings.

**Figure 9:**
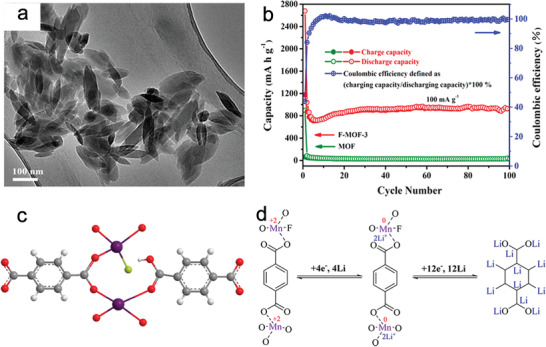
a) SEM image, b) cycling performance, c) local structure, and d) electrochemical reaction mechanism of the as‐prepared F‐doped Mn‐MOF material. Reproduced with permission.^[^ ^97^ ^]^ Copyright 2017, American Chemical Society.

To further improve the lithium storage performance, ligand modification or combination with other materials has been used. By replacing some of the benzenedicarboxylate linkers with F^−^, the fluoride ions interact with Li^+^, which promotes Li^+^ transfer. An F‐doped Mn‐based NMOF with fusiform morphology was fabricated (**Figure** **9**a). The reduction/oxidation reaction of Mn^2+^→Mn^0^/Mn^0^→Mn^2+^ and lithiation/delithiation of unsaturated carbon in organic ligands occurred during discharge/charge, thereby delivering a higher reversible capacity, rate capability, and coulombic efficiency than pure Mn‐MOF (Figure 9b–d).^[^ ^97^ ^]^ Lan et al. introduced polyoxometalate (POM) to a Cu‐MOF to realize excellent electrochemical performance (1723 mAh g^−1^ at 200 mA g^−1^, 1072 mAh g^−1^ at 1000 mA g^−1^ after 400 cycles).^[^ ^98^ ^]^ Subsequently, they encapsulated ionic liquids into POM‐based MOFs to facilitate conductivity, which led to superior lithium storage performance.^[^ ^99^ ^]^ Other methods have also been used to improve the electrochemical performance of NMOFs, such as anchoring NMOFs on few‐layer black phosphorus/reduced graphene oxide (MOF thickness: ≈6 nm, particle size: 200 nm),^[^ ^100^, ^101^ ^]^ integrating NMOFs with SnO_x_ quantum dots (MOF size: 50 nm).^[^ ^102^ ^]^

NMOFs can also be used as cathode materials in LIBs. Yamada et al. directly used MIL‐101(Fe) as the cathode for LIBs, which showed a discharge capacity of 72 mAh g^−1^ at 0.2 C after 100 cycles.^[^ ^103^ ^]^ Chen et al. fabricated nanosheets of a conductive Cu‐based MOF (Cu_3_(HHTP)_2_) from a 2,3,6,7,10,11‐hexahydroxytriphenylene (HHTP) linker (conductance: 2.1 × 10^−1^ S cm^−1^). The unique nanosheet structure (sheet size: 20–40 nm) was believed to permit fast storage and diffusion of Li ions, which resulted in a reversible discharge capacity of 95 mAh g^−1^ and a highly stable redox cycling performance up to 500 cycles at a high current density of 20 C. Disappointingly, the Cu_3_(HHTP)_2_ nanosheets suffered from fast capacity loss, probably due to irreversible changes in copper redox centers during charging (Cu^+^→Cu^2+^).^[^ ^104^ ^]^ Although some progress has been achieved, the lower specific capacities and energy densities compared with commercially‐available intercalated Li compounds make NMOFs less studied as LIB cathodes compared with anode materials.

NMOFs have also been investigated for electrolyte design and separator modification, not only for LIBs but also for LSBs, Li‐O_2_ batteries, and sodium‐based batteries, which will be discussed in Section 3.3.

As demonstrated above, there are some remaining issues, such as largely irreversible capacity decay, incomplete electrode reactions, and poor cycling stability. Despite these shortcomings, NMOFs show bright prospects as LIBs electrodes. The small particle size of NMOF‐based anodes can shorten the Li^+^ diffusion distance and time (*L* = (*Dt*)^1/2^ (where *L* represents the diffusion distance, *D* represents the diffusion coefficient, and *t* represents the time), therefore improving the specific capacity and rate performance; however, the irreversible formation or regeneration of the original NMOF may decrease its recyclability. NMOF‐based cathodes have received less attention than anodes, mainly due to the following reasons: 1) Similar to anodes, the irreversible structure changes can cause a poor cycle life; 2) the mismatched voltage plateau tends to cause a poor energy density; 3) the close packing of smaller NMOFs can result in a lower volumetric energy density than microsized cathode materials used in commercial LIBs. Combined with the demerits of NMOFs‐based anodes and cathodes, future directions should identify new NMOF candidates that possess robust frameworks with open channels to facilitate rapid Li^+^ transport without damaging the NMOF structure. The materials should also possess multi‐valence metal ions and/or organic ligands with abundant functional groups to strongly anchor Li^+^, and organic ligands with high redox potentials to enhance the overall voltage plateau of NMOFs.

#### 3.1.2 Lithium‐Sulfur Batteries

Rechargeable LSBs are ideal next‐generation energy storage and conversion devices because of their theoretical lithium storage, which exceeds the limit of traditional LIBs. This is attributed to many advantages because of the use of sulfur as the cathode, including its high theoretical capacity (1672 mAh g^−1^) and energy density (2600 Wh kg^−1^), its abundance in the Earth's crusts and its environmental friendliness;^[^ ^105^ ^]^ however, there are still several issues that prevent its commercialization. First, volume changes during discharge–charge can pulverize active materials and cause rapid capacity fading. Second, electrochemically and ionically insulating sulfur and its discharge product (Li_2_S) significantly limit the rate performance. Third, the shuttle effect of dissoluble polysulfides (Li_2_S_n_, *n* = 4–8) decreases the active mass utilization during discharge and greatly decreases the coulombic efficiency.^[^ ^106^ ^]^ If these challenges are addressed, NMOFs with high surface areas and sufficient spaces will provide an attractive platform for designing promising cathode host materials for LSBs. In detail, the large pore volume and tunable porosity of NMOFs can provide spaces to encapsulate sulfur, polysulfides, and intermediates. The nanoscale dimensions of MOFs can prevent the pulverization of the sulfur cathode and shorten the transport pathways for electrons and Li ions to obtain high capacity and rate performance.

**Figure 10:**
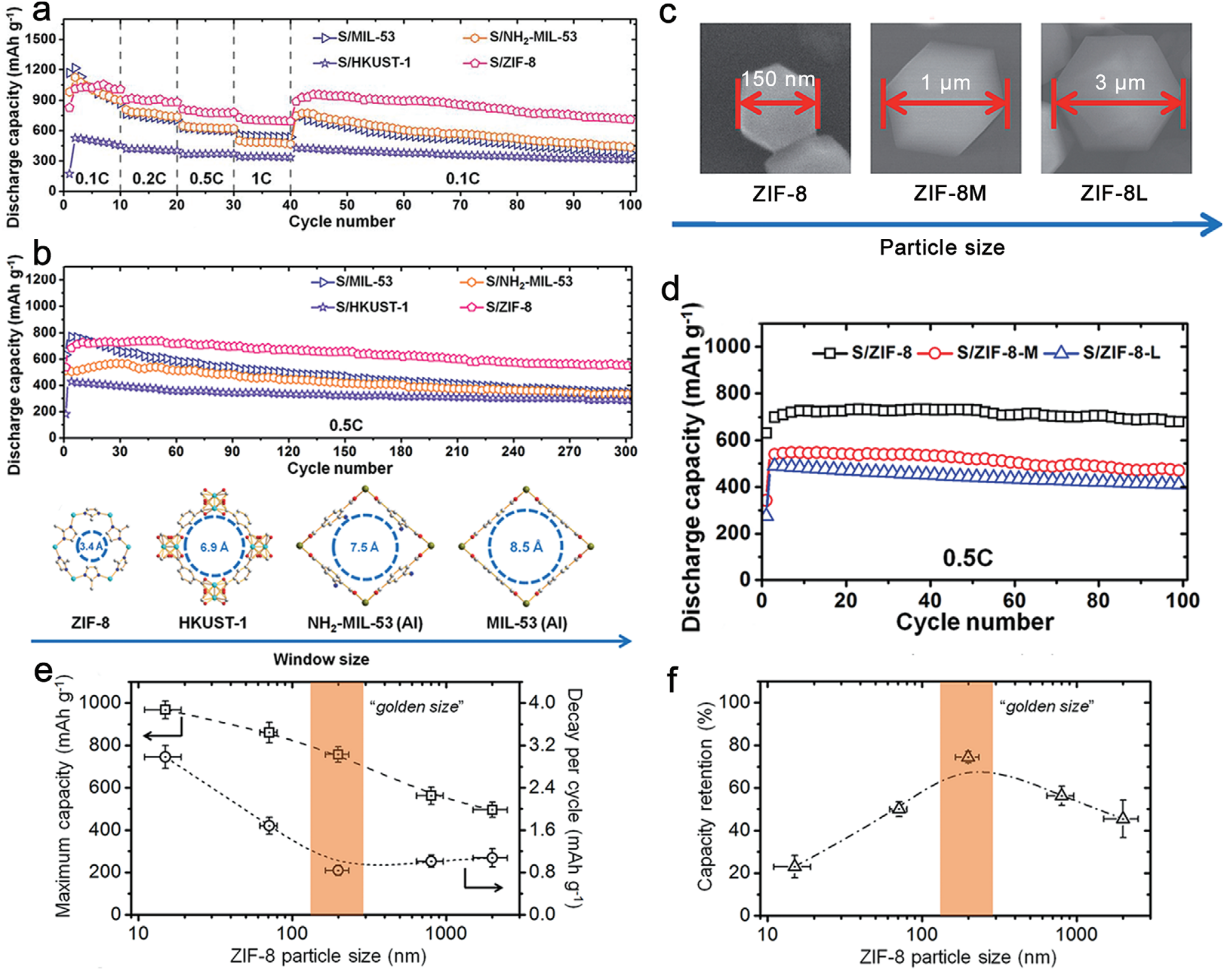
a) Rate capabilities of S/MOFs. b) Cycling performance of S/MOFs at 0.5 C (top) and schematic of the largest apertures of the four MOFs (down). c) Size comparison among ZIF‐8 crystals. d) The influence of ZIF‐8 size on the specific capacity. Reproduced with permission.^[^ ^107^ ^]^ Copyright 2014, The Royal Society of Chemistry. The relationship of e) maximum capacity and average decay per cycle and f) capacity retention over 250 cycles with the particle size of ZIF‐8. Reproduced with permission.^[^ ^67^ ^]^ Copyright 2015, The Royal Society of Chemistry.

Zhou et al. designed a nanoscale ZIF‐8 embedded with sulfur molecules inside pores as a cathode catalyst for LSBs.^[^ ^107^ ^]^ Normally, the sulfur loading amount in the ZIF‐8 nanocrystals is ≈50 wt%, which is higher than those of former MOF materials.^[^ ^108^, ^109^ ^]^ When used as a cathode, sulfur@nanoscale ZIF‐8 exhibited a high capacity of 1055 and 710 mAh g^−1^ (based on sulfur) at current densities of 100 and 1000 mA g^−1^, respectively. In addition, the capacity loss after 300 cycles at 500 mA g^−1^ current density was only 0.08%, demonstrating remarkable long‐term cyclability (**Figure** **10**a). To clarify the structure‐property relationships of MOFs and determine the influence of the MOF particle size on its electrochemical performance, MOFs with different structural features and ZIF‐8 microparticles (0.8–1.2 µm, 2.5–3.5 µm) were synthesized for comparison (Figure 10b–d).^[^ ^110^ ^]^ The results indicated that immobilization would be substantially weakened for MOFs with window sizes larger than the diameter of an S_8_ ring (6.9 Å), which resulted in rapid capacity decay during cycling. Besides, ZIF‐8 with a smaller particle size was more likely to achieve a high capacity as confirmed in ref. ^[^ ^67^ ^]^, where the electrochemical performance of ZIF‐8 crystals with different particle sizes from 15 nm, 70 nm, 200 nm, 800 nm, and 2 µm was compared. The results showed that sulfur utilization increased monotonically with decreasing particle size, and the optimal capacity retention was obtained by ZIF‐8 with a particle size of 200 nm (Figure 10e,f).

Besides the immobilizing effect of their internal spaces, NMOFs might also limit the dissolution of polysulfide intermediates in electrolytes. Cai et al. reported a dual functional cage‐like MOF (Cu‐MOF) as a sulfur host in LSBs.^[^ ^111^ ^]^ The nitrogen atoms of the 2,4,6‐tris(3,5‐dicarboxylphenylamino)‐1,3,5‐triazine ligand acted as Lewis basic sites, and the exposed metal Cu(II) Lewis acidic sites interacted with polysulfide anions (S_x_^2−^), which increased the cycling stability (500 cycles).

Post‐modification of NMOFs or their organic linkers is another essential strategy to trigger bonding interactions with polysulfides. Zhang et al. introduced thiol (—SH) groups into a nanoscale UiO‐66 framework (50–200 nm) to covalently immobilize sulfur onto the MOF via the in situ transformation of —SH groups at 185 °C under an argon atmosphere.^[^ ^112^ ^]^ The as‐formed sample improved the cycling stability for up to 900 cycles with a high capacity retention of 80.19%. Yin et al. modified a nanoscale ZIF‐67 system using tannic acid (TA) to rationally tune its polarity, hydrophilicity, and porous structure. The H^+^ released from TA cleaved the Co—N bonds and promoted the formation of new N—H bonds, which enhanced the chemical immobilizing effect for sulfur/polysulfides and improved the cycling performance and rate capability than that of pristine ZIF‐67.^[^ ^113^ ^]^

Unfortunately, the electrochemical performance of NMOFs has been severely hindered by their poor electrical conductivity. This has motivated the complexation of NMOFs with conductive inorganic materials or polymers, including graphene, carbon nanotubes, and polypyrrole (PPy). Yan et al. chemically grafted nanoscale UiO‐66 with an average diameter of 80 nm onto PPy nanotubes to improve the electron transfer paths and suppress the shuttle effect, which resulted in excellent electrochemical durability.^[^ ^114^ ^]^ Similarly, Pang et al. fabricated hollow ZIF‐67 within PPy to form a core‐shell structure, in which the hollow structure buffered the volume expansion of the battery, while the PPy coating improved the electroconductivity.^[^ ^115^ ^]^ The synergistic effect between the two components improved the specific capacity and life span. Tang et al. interlaced carbon nanotubes into UiO‐66 to construct a defective MOF architecture to obtain LSBs with long cycling lifetimes.^[^ ^116^ ^]^

In summary, NMOFs have multiple advantages, including small crystal sizes, adjustable apertures, and facile functionalization of the open framework, which make them promising host materials for storing sulfur and anchoring dissolved polysulfides when used in LSBs. Smaller MOFs can shorten the internal diffusion lengths of Li^+^/e^−^, which can facilitate the utilization of sulfur, that is, more sulfur atoms become electrochemically active due to more Li^+^/e^−^ per sulfur atom. This enhances the reversible capacity, similar to LIBs. Nevertheless, the small size does not significantly impact the cyclability of the structure, as verified by the examples described above; therefore, crystal size is generally a secondary factor of a MOF host material. Only when it affects the performance of a certain MOF should the size be precisely tailored.

#### 3.1.3 Lithium‐Oxygen Batteries

Li‐O_2_ batteries possess a higher theoretical energy density (3505 W h kg^−1^) than LIBs and LSBs, and are thus considered the most promising alternative to state‐of‐the‐art batteries.^[^ ^105^ ^]^ However, the sluggish charge transfer during discharge–charge gives rise to poor cyclability, low round‐trip efficiency, and unsatisfactory rate performance, thus hindering their applications. For this reason, cathode catalysts with high oxygen adsorption activities are urgently needed. NMOFs, with large surface areas and pore volumes, tunable pore sizes, and open channels, show more favorable oxygen adsorption and faster electron and oxygen transports. Meanwhile, the open metal sites of NMOFs promote reactions between NMOFs and small molecules, making NMOFs suitable for use as cathode catalysts for Li‐O_2_ batteries.

**Figure 11:**
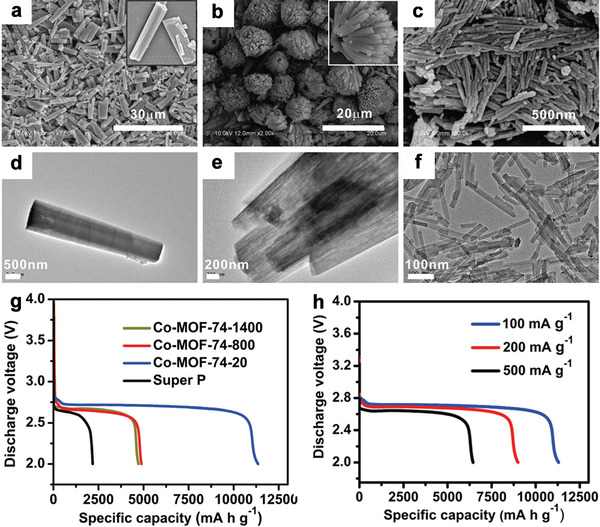
a–c) SEM images of Co‐MOF‐74‐1400, Co‐MOF‐74‐800, and Co‐MOF‐74‐20 crystals. d–f) TEM images of Co‐MOF‐74‐1400, Co‐MOF‐74‐800, and Co‐MOF‐74‐20 crystals. g) Discharge curves of Li‐O_2_ batteries at a current density of 100 mA g^−1^ for MOF‐Super P composite electrodes or pure Super P electrodes. h) Discharge profiles of Li‐O_2_ batteries with Co‐MOF‐74‐20 based at various current densities. Reproduced with permission.^[^ ^43^ ^]^ Copyright 2017, The Royal Society of Chemistry.

In 2014, Li et al. reported the first MOF cathode catalysts for Li‐O_2_ batteries by.^[^ ^117^ ^]^ Subsequently, a microsized Ni‐MOF‐74 was used as a cathode for Li‐O_2_ batteries and displayed a high capacity of 9000 mAh g^−1^ and excellent cyclability, which was associated with its inherent structural characteristics, which guaranteed the free transfer of O_2_ and effective contact between the electrolyte and catalytic sites.^[^ ^118^ ^]^ However, the influence of the MOF crystal size on its electrochemical performance improvement was not revealed until recently. Li et al. synthesized a series of 1D Co‐MOF‐74 nanorods with diameters of 20, 800, and 1400 nm by adjusting the solvent mixture ratio, together with introducing additional modulators (**Figure** **11**).^[^ ^43^ ^]^ The results showed that decreasing the particle size of Co‐MOF‐74 indeed improved the Li‐O_2_ battery performance. Particularly, the Co‐MOF‐74‐20 catalyst showed the highest initial discharge capacity of 11 350 mAh g^−1^ at 100 mA g^−1^, surpassing Co‐MOF‐74‐800 and Co‐MOF‐74‐1400, which both displayed discharge capacities below 5000 mAh g^−1^. An optimized rate performance with a capacity of 6440 mAh g^−1^ at 500 mA g^−1^ was also observed for Co‐MOF‐74‐20, which was mainly ascribed to the abundant unsaturated active sites, smaller particle size, and inherent defects. Ultrathin 2D MOFs also have high O_2_ accessibility, open catalytic active sites, and large surface areas. Ma et al. reported a series of 2D MOFs and investigated their performance as cathode catalysts for Li‐O_2_ batteries.^[^ ^119^ ^]^ It was revealed that the 2D Mn‐MOF delivered a higher initial discharge capacity (9464 mAh g^−1^) and superior cyclability (over 200 cycles) than Co‐MOF and Ni‐MOF. This was mainly due to the higher band gap of Mn‐MOF than Co‐ and Ni‐MOFs, which demonstrated the critical influence of electrons in Mn‐MOF on the ORR and OER. Additionally, the unique 2D structure also exposed plentiful unsaturated active sites and promoted rapid electron transfer, allowed rapid mass transport, and ensured close contact between MOFs and the electrolyte, which all contributed to the excellent electrochemical performance.

Although NMOFs have significantly progressed as Li‐O_2_ battery cathodes, they require further improvements. According to aforementioned studies, the dominant reaction mechanism of NMOF involves catalytic active sites, including unsaturated variable‐valence metal ions and inherent defects of organic ligands, which can promote the ORR and OER and enhance the electrochemical performance. However, irreversible capacity decay due to the formation of insulating Li_2_O_2_ discharge product, poor cyclability, or NMOF framework collapse means that the materials are still far from practical application. Thus, future directions should focus on designing and constructing NMOFs with plentiful redox‐active sites, low overpotentials, large surface areas and apertures, and robust structures to accommodate generated Li_2_O_2_ and facilitate its reversible decomposition.

### 3.2 Other Batteries

Some other batteries, including sodium‐ion batteries (SIBs) and potassium‐ion batteries (PIBs), have been investigated for potential applications in energy storage and conversion technologies, due to the low cost and natural abundance of sodium and potassium resources. Considerable efforts have been made to adapt the NMOFs developed for LIBs systems for use in SIBs, especially electrode materials. Bao et al. demonstrated the high performance of 2D cobalt‐based MOF (Co‐HAB, HAB = hexaaminobenzene) with stable, accessible, dense active sites, and a high conductivity of 1.57 S cm^−1^ as the anode to provide high‐performance sodium storage.^[^ ^120^ ^]^ The Co‐HAB stored three electrons and Na^+^ per HAB in the organic electrolyte, and delivered a high specific capacity, stable life span, and rate capability. Because of the similar physicochemical properties of K^+^, Na^+^, and Li^+^ and their abundant sources, PIBs are also considered promising LIBs alternatives. Chen et al. reported a layered vanadium‐based NMOF (K_2_[(VO)_2_(HPO_4_)_2_(C_2_O_4_)], KVPC‐S) with a sheet thickness of 100–300 nm and large interplanar lattice spacing,^[^ ^121^ ^]^ which exhibited highly reversible K^+^ extraction/insertion and good cycling stability (200 cycles) as a cathode material for PIBs. They also found that KVPC‐S MOF decreased the voltage plateau to 4.1 V. NMOFs have also been applied in zinc‐ion batteries and lithium‐selenium batteries, but have received less attention.^[^ ^122^, ^123^ ^]^

### 3.3 Separator and Electrolyte Systems

As essential components of rechargeable batteries, especially LIBs and LSBs, separator and electrolyte systems inhibit the offering of internal ion conduction routes and the short circuits caused by the generation of lithium dendrites on the electrode surface.^[^ ^124^, ^125^ ^]^ Because of their high porosity, tunable pore sizes, high surface area, and nanoscale morphology, NMOF‐based membranes can be used for uniform Li‐ion plating and to restrict the reverse ion shuttling inside the electrolyte via the formation of a physical barrier.^[^ ^125^ ^]^ For instance, by fully utilizing metal sites in the NMOF to anchor anions in the liquid electrolyte of LIBs, the Li^+^ transfer number and efficiency were greatly promoted, which prolonged the Li anode stability.^[^ ^126^ ^]^ Comparably, Li et al. modified ZIF‐67 on a polypropylene separator by a direct coating strategy to improve capacity retention and circulation stability of a battery.^[^ ^126^ ^]^ The pore structure and the large specific area of ZIF‐67 prepared in methanol (ZIF‐67‐CH_3_OH) showed improved electrolyte wettability compared with ZIF‐67 obtained in water (ZIF‐67‐H_2_O). This was because of the slower migration of Li^+^ inside the electronegative micropores of ZIF‐67‐H_2_O, which transported the missing H_2_O solvent away from Co nodes.

**Figure 12:**
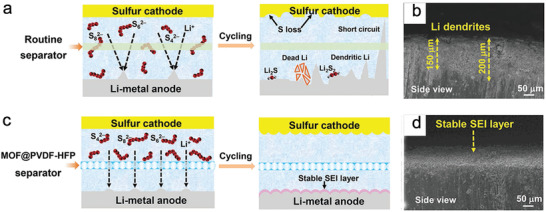
Schematic and typical SEM images for LSBs with routine a,c) and MOF@PVDF‐HFP b,d) separators. Reproduced with permission.^[^ ^127^ ^]^ Copyright 2018, Wiley‐VCH.

NMOFs have frequently been used as separators in LSBs, performing well as blocking interlayers to inhibit polysulfide migration from the cathode to anode. Zhou et al. used a flexible PVDF‐HFP membrane embedded with HKUST‐1 nanoparticles (MOF@PVDF‐HFP) as a separator for a high‐performance LSB with an ultralong cycle life (2000 cycles) and ultralow capacity loss (0.015% per cycle).^[^ ^127^ ^]^ The narrow and uniform pore size of NMOF particles inside the separator simultaneously suppressed polysulfides shuttling and Li dendrite growth (**Figure** **12**). In another case, Lu et al. reported a UiO‐66 (particle size: 400 nm) modified polypropylene separator that acted as both a physical and chemical barrier to prevent the shuttle effect in LSBs. The LSB delivered an enhanced circulation capability with a specific capacity of 720 mAh g^−1^ at a current density of 0.5 C for 500 cycles.^[^ ^128^ ^]^ Additionally, other NMOFs such as sulfonated UiO‐66, Cu‐TCPP nanosheets (TCPP = 5,10,15,20‐tetrakis(4‐carboxyphenyl) porphyrin), conductive Ni_3_(HITP)_2_ (HITP = 2,3,6,7,10,11‐hexaiminotriphenylene), and Ce‐MOF (350 nm) have also been applied in LSBs as separators and have enhanced the capacity retention and cycling stability.^[^ ^129^, ^130^, ^131^, ^132^ ^]^ Recently, Kim et al. coated multi‐walled carbon nanotubes with small Ni‐based MOF particles onto a polyethylene separator to increase the discharge capacity and cycling stability.^[^ ^133^ ^]^ Detailed analyses revealed that the Lewis acid Ni^2+^ sites of Ni‐MOF strongly interacted with the polysulfide anions, which prevented polysulfide migration.

**Figure 13:**
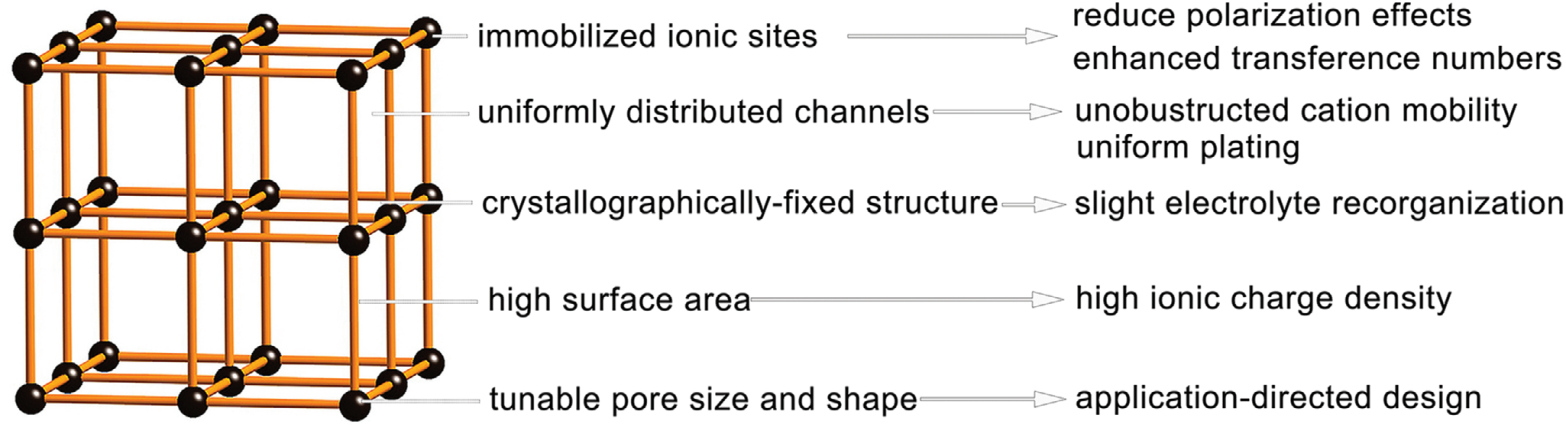
The advantages of NMOF solid‐state electrolytes. Reproduced with permission.^[^ ^133^ ^]^ Copyright 2019, The Royal Society of Chemistry.

Most battery systems use flammable liquid‐based electrolytes. Alternatively, solid‐state electrolytes (SSEs) have emerged to enhance the safety by removing hazardous liquid materials. They may also anchor charge‐balancing anions and provide battery system with a stable potential window.^[^ ^134^, ^135^ ^]^ NMOFs can be directly utilized, or they may act as additives in SSEs due to their diverse advantages (**Figure** **13**). For instance, Dong et al. reported an SSE based on 50 nm ZIF‐8 particles, which showed high electrochemical performance induced by high ionic conductivity and Li^+^ transfer number.^[^ ^136^ ^]^ Li and coworkers blended different MOFs (UiO‐66, MIL‐101‐NH_2_, HKUST‐1) with carbonate electrolytes to adjust the stability of Li anode during cycling and found that the morphology, particle size, and porosity greatly impacted the polarization retention capability.^[^ ^137^ ^]^ In particular, UiO‐66 delivered the best Li anode plating/stripping for up to 1400 h with improved kinetics and without serious degradation of its voltage polarization. An ionic liquid incorporated with Ni‐BTC MOF was also added into a PVdF‐HFP‐based polymer electrolyte to optimize its ionic conductivity (6.5 × 10^−3^ S cm^−1^) and electrochemical stability.^[^ ^138^ ^]^

As seen, NMOFs have shown great advantages than other traditional porous carbon‐based materials and inorganic oxide materials when used in electrolyte and separator of the lithium‐based batteries. For NMOF‐based electrolytes, on one hand, the adjustable pore sizes make NMOF membranes easily match with electrolyte anions sizes and confine their transport. This promotes the high and homogeneous mobility of Li^+^ ions, preventing the growth and Li dendrites and achieving stable Li deposition. On another hand, they serve as ionic sieves to allow ion hopping of small size ions (e.g., Li^+^) while confining the migration of ions with large scale. In addition, they also present the feasibility and potential of a new approach for developing new solid electrolyte materials for LIBs, thereby expanding the application of LIBs. For NMOF‐based separators, LSBs and Li‐O_2_ batteries are preferred. They can not only effectively restrain the adverse shutting of polysulfides for accomplishing long cycle life LSBs, but also be employed to develop dual‐mediator strategies for excellent electrochemical performance Li‐O_2_ batteries.

## 4 Conclusions and Perspectives

Research into NMOFs applications has seen explosive growth recently, especially for energy storage and conversion systems such as batteries. This is attributed to their many advantages, which improve electrochemical processes compared with bulk MOFs, including shorter diffusion pathways for faster and more effective transport of guest molecules, more accessible active sites for surface reactions, and more opportunities to be functionalized with other inorganic components. Because of this, various strategies have been developed to fabricate NMOFs to meet the requirements of many fields.

Since many advances have been made on the synthesis and applications of NMOFs, it is necessary to summarize general regulation strategies and their mechanisms. The properties of NMOFs have been tailored by synthesis parameter regulation (concentration, reactant type, reaction conditions), coordination adjustment (acids, bases, inorganic salts, surfactants), and other strategies, such as liquid exfoliation, and salt‐template confinement. These methods work by either regulating the coordination reaction equilibrium between metal ions or nodes with organic linkers, or by competitively coordinating with additives against organic linkers. For the practical applications of NMOFs in batteries, numerous advances have been achieved and were summarized here, especially for LIBs, LSBs, and Li‐O_2_ batteries, in which NMOFs have served as electrodes, separators, and electrolytes.

Despite the above achievements, there are still fundamental that must be addressed. For example, many examples have confirmed that downsizing MOFs is realized through the synergistic cooperation of multiple parameters, rather than a single one, and each parameter is theoretically critical for NMOF preparation. To further advance this field, we propose that more attention should be paid to the following aspects: 1) The targeted construction of NMOFs will rely on determining the relationship between the intrinsic structures of MOFs and their final nanomorphologies, as well as the impact of synthesis conditions on the product morphologies. Future development may investigate the large scale production of NMOFs using highly effective solution‐based direct precipitation methods. 2) The exploration of new NMOFs is a primary task since developing NMOFs with various compositions and structures is fundamental to their future applications. 3) For broader applications and NMOFs with improved performances, the following strategies are expected: designing and constructing conductive NMOFs or improving the electrical conductivity of NMOFs by infiltrating redox‐active and conjugated guest molecules; synthesizing multivariate NMOFs with more functional groups through chemical post‐modification; combining with conductive additives such as graphene, carbon nanotubes, polyaniline, etc. 4) Similar to pristine MOFs, the electrochemical energy storage and degradation mechanism of NMOFs as electrodes might be clarified using in situ characterization techniques such as HEXRD, XAFS, and HAXPES.

This review highlighted the elements for regulating NMOFs, as well as their applications as battery electrodes, separators, and electrolytes. This may provide a useful reference for future research to precisely regulate the crystalline size and morphology of NMOFs for nanotechnology applications.

## Conflict of Interest

The authors declare no conflict of interest.
